# Sir William Osler

**Published:** 1919-12

**Authors:** 


SIR WILLIAM OSLER, BART., M.D., F.R.S., F.R.C.P.

As we go to press we hear with deepest regret the news of Osler's death, at the age of 70. His life was a remarkable instance of what one man may achieve. His three professorships at McGill, Johns Hopkins and Oxford are proof enough that Osler was cast in no ordinary mould. His text-book found a wider public than any medical work since Galen. The wisdom of his public utterances won universal acknowledgment. Yet what we shall chiefly miss him for is his warm-hearted friendship. In all history there has been no one who was the personal friend of so many of his fellows. Osler's greatest gift was his capacity for interesting himself in the doings and interests of others. Some are accounted leaders because they attract a host of infatuated devotees ; it was otherwise with Osler, he was not made of the stuff that absorbs adulation. His greatness lay in what he gave so freely : his encouragement, his sympathetic understanding and his friendship. We do not mean to belittle his own contributions to medicine. We hold in higher esteem the unique position he occupied as a midwife of true learning.

